# Corneal confocal microscopy differentiates inflammatory from diabetic neuropathy

**DOI:** 10.1186/s12974-021-02130-1

**Published:** 2021-04-08

**Authors:** Michael Fleischer, Inn Lee, Friedrich Erdlenbruch, Lena Hinrichs, Ioannis N. Petropoulos, Rayaz A. Malik, Hans-Peter Hartung, Bernd C. Kieseier, Christoph Kleinschnitz, Mark Stettner

**Affiliations:** 1 Department of Neurology and Center for Translational and Behavioral Neurosciences (C-TNBS), University Hospital Essen, University Duisburg-Essen, Essen, Germany; 2 Department of Neurology, Medical Faculty, Heinrich-Heine-University, Düsseldorf, Germany; 3 Department of Cardiology, University Hospital Essen, University Duisburg-Essen, Essen, Germany; 4 Institute of Cardiovascular Science, Faculty of Medical and Human Sciences, University of Manchester, Manchester, UK; 5 Weill Cornell Medicine-Qatar, Educator City, Doha, Qatar; 6 Brain and Mind Centre, University of Sydney, Sydney, Australia; 7 Medical University Vienna, Vienna, Austria; 8 Department of Neurology, Palacky University, Olomouc, Czech Republic

**Keywords:** Chronic inflammatory demyelinating neuropathy, corneal confocal microscopy, Diabetes mellitus

## Abstract

**Background:**

Immune-mediated neuropathies, such as chronic inflammatory demyelinating polyneuropathy (CIDP) are treatable neuropathies. Among individuals with diabetic neuropathy, it remains a challenge to identify those individuals who develop CIDP. Corneal confocal microscopy (CCM) has been shown to detect corneal nerve fiber loss and cellular infiltrates in the sub-basal layer of the cornea. The objective of the study was to determine whether CCM can distinguish diabetic neuropathy from CIDP and whether CCM can detect CIDP in persons with coexisting diabetes.

**Methods:**

In this multicenter, case-control study, participants with CIDP (*n* = 55) with (*n* = 10) and without (*n* = 45) diabetes; participants with diabetes (*n* = 58) with (*n* = 28) and without (*n* = 30) diabetic neuropathy, and healthy controls (*n* = 58) underwent CCM. Corneal nerve fiber density (CNFD), corneal nerve fiber length (CNFL), corneal nerve branch density (CNBD), and dendritic and non-dendritic cell density, with or without nerve fiber contact were quantified.

**Results:**

Dendritic cell density in proximity to corneal nerve fibers was significantly higher in participants with CIDP with and without diabetes compared to participants with diabetic neuropathy and controls. CNFD, CNFL, and CNBD were equally reduced in participants with CIDP, diabetic neuropathy, and CIDP with diabetes.

**Conclusions:**

An increase in dendritic cell density identifies persons with CIDP. CCM may, therefore, be useful to differentiate inflammatory from non-inflammatory diabetic neuropathy.

**Supplementary Information:**

The online version contains supplementary material available at 10.1186/s12974-021-02130-1.

## Background

Immune-mediated neuropathies are a heterogeneous group of conditions mediated by inflammatory processes resulting in impaired sensation and muscle weakness which impose a significant burden of disease [1]. The most common immune-mediated neuropathy is chronic inflammatory demyelinating polyneuropathy (CIDP), with a prevalence of around 1–2/100,000 people [2]. The diagnosis of CIDP relies primarily on nerve conduction studies; however, diagnostic challenges can lead to a considerable degree of mis- and under-diagnosis [3, 4]. In some cases, neurophysiology may not clearly differentiate CIDP from other demyelinating neuropathies, such as demyelinating diabetic neuropathy or demyelinating hereditary neuropathies [5]. Furthermore, epidemiological data suggest an increased prevalence of CIDP among individuals with diabetes [6]. This, coupled with the difficulty of diagnosing CIDP in persons with diabetic neuropathy, may preclude the application of timely and specific treatment options such as intravenous immunoglobulins for CIDP [7].

Corneal confocal microscopy (CCM) is a relatively rapid non-invasive ophthalmic imaging technique that is reproducible [8, 9] and well tolerated [10]. This technique has demonstrated nerve fiber loss and provided good diagnostic utility for diabetic neuropathy in larger cohorts [11–13], comparable to intraepidermal nerve fiber density [14] and also has shown predictive utility for the development of diabetic neuropathy [15]. CCM also identifies corneal nerve loss in a range of peripheral neuropathies including idiopathic small fiber neuropathy [16], Charcot-Marie-Tooth disease type 1A [17], HIV neuropathy [18], chemotherapy-induced peripheral neuropathy [19], amyloid neuropathy [20], and Friedreich’s ataxia [21].

There is currently no non-invasive technique that can act as a surrogate measure of ongoing inflammation to assess disease progression or treatment response and dose adjustment of therapies. Experimental data indicate that the presence of dendritic cells and their contact with the sub-basal nerve plexus may trigger nerve fiber damage [22]. More recently, we have shown an increase in corneal dendritic cells in individuals with CIDP and suggested that this may help to stratify CIDP subtypes, clinical course, and disease activity [23]. In a prospective study of 17 individuals with CIDP who were followed over 18 months, the presence of > 30 cells/mm^2^ at baseline identified clinical progression with a sensitivity and specificity of 100% [24]. Moreover, in one person with anti-neurofascin-155 neuropathy, treatment with rituximab was associated with a reduction in the serum antibody titer with a clinical and electrophysiological improvement and reduction of corneal inflammatory cell infiltrates [25].

The present study was undertaken to assess whether CCM can differentiate CIDP from diabetic neuropathy and whether it can detect CIDP in persons with coexisting diabetes.

## Methods

### Study design and participants

The study was performed in accordance with the principles of the Declaration of Helsinki, and the local Ethics Committees approved the study plan (Ethics Committee University of Essen, #16-7289-BO, North Manchester Ethics Committee). Participants who provided written informed consent were included. All participants were over 18 years of age. A total of 55 patients with CIDP, 58 patients with type 2 diabetes and 58 healthy controls were investigated. Within the CIDP group, 10 patients also had type 2 diabetes, and 11 had monoclonal gammopathy of undetermined significance (MGUS+), (Table 1). CIDP was diagnosed according to the European Federation of Neurological Societies/Peripheral Nerve Society criteria [26]. Patients with diabetes underwent assessment of the neuropathy symptom profile (NSP) and modified neuropathy disability score (NDS) to assess pinprick, vibration perception, temperature sensation and ankle reflexes. Nerve conduction studies (NCS) were undertaken and diabetic neuropathy was defined according to the Toronto consensus, which requires the presence of symptoms (abnormal NSP) or signs of neuropathy (NDS > 2) and abnormal peroneal nerve conduction velocity (PMNCV < 40 m/s) and the patients were subdivided into those with diabetic neuropathy (*n* = 28) and those without (*n* = 30) [27].

**Table 1:** Subgroups and demographics of participants.

Cohort (total ***n*** = 171)	*n*	Age	Sex Male (%)	Female (%)
CIDP	**55**	**58 ± 12.7**	**60**	**40**
CIDP + diabetes	10	55 ± 8.3	90	10
CIDP −diabetes	45	59 ± 12.3	53	47
CIDP + MGUS	11	58 ± 9.3	66	34
Diabetes	**58**	**52 ± 16.7**	**47**	**53**
Diabetes +to	28	61 ± 10.8	43	57
Diabetes −to	30	43 ± 16.6	47	53
Healthy controls	**58**	**49 ± 15.3**	**34**	**66**

*Abbreviations*: *CIDP* chronic inflammatory demyelinating polyneuropathy, *MGUS* monoclonal gammopathy of undetermined significance, *DN* diabetic neuropathy, patients with (+to) and without (−to) neuropathy according to the Toronto criteria

Patients with CIDP who were positive for anti-MAG antibodies were excluded. In the healthy control group, a full blood workup and clinical, neurological, and neurophysiological examination were performed to exclude neuropathy. Patients and controls were recruited from the Department of Neurology, University Hospital of Essen, Germany, and from the Centre for Endocrinology and Diabetes, University of Manchester, UK.

### Corneal confocal microscopy

Corneal images were captured using a Heidelberg Retina Tomograph (HRT III, Rostock Cornea Module, Heidelberg Engineering, Heidelberg, Germany). Corneal integrity was confirmed by slit-lamp examination. Local anesthetic (0.4% benoxinate hydrochloride) was used to anesthetize the eye, and a drop of Viscotears Liquid Gel was used between the lens and the disposable lens cover. CCM is a corneal contact technique which has a very low risk for corneal injury or keratitis; however, none of our patients developed any of these complications. Four scan cycles were conducted across the entire depth of the central cornea, especially the sub-basal nerve layer. At least 15 images per patient, meeting established quality criteria were analyzed [10]. Automated corneal nerve quantification was undertaken using established software (ACCMetrics Image Analysis tool v1.1, University of Manchester, UK) to evaluate the following: corneal nerve fiber density (CNFD; no./mm^2^), corneal nerve fiber length (CNFL; mm/mm^2^), and corneal nerve branch density (CNBD; major no./mm^2^). Cell quantification was performed in a blinded manner without knowledge of patient diagnosis using ImageJ software (version 1.41, National Institutes of Health, USA). Cells were classified as dendritic cells with fiber contact (DCF), dendritic cells in the periphery without fiber contact (DCP), non-dendritic cells with fiber contact (NCF), or non-dendritic cells in the periphery without fiber contact (NCP), as described previously [23]. Dendritic and non-dendritic cells were counted per mm^2^. F/mm^2^ comprises all cells/mm^2^ with fiber contact (DCF and NCF), whereas P/mm^2^ combines all cells per mm^2^ without fiber contact (DCP, NCP).

### Statistical analysis

All data are presented as mean, standard error of the mean, and *P* values, which were calculated using GraphPad Prism software version 9.0 (GraphPad Software, Inc., La Jolla, CA, USA). Differences between groups were assessed using Kruskal-Wallis one-way analysis of variance with Dunn’s multiple comparison post hoc tests, after analyzing for parametrical distribution with Shapiro-Wilk test. A *P* value < 0.05 was considered to be significant (*< 0.05, **< 0.01, and ***< 0.001). Specificity, sensitivity, and positive predictive value were calculated for distinguishing CIDP from DN and healthy controls with the parameter DCP and DCF by using the lower *z* value of two times the SEM from the median as the cut-off value.

## Results

The mean age of patients with CIDP was 58 ± 12.7 years and 60% were males, compared to the group of patients with diabetes who had a mean age of 52 ± 16.7 years and 47% were males. The groups and subgroups (CIDP ± diabetes, CIDP ± MGUS, diabetes ± to) were matched with regard to demographic characteristics and did not differ significantly. The mean age of healthy controls was 49 ± 15.3 years and 58% were males (Table 1). The potential effect of age or sex as a confounding factor was examined by multiple regression analysis (method enter) and logistic regression analysis. Neither factor was shown to have any influence on relevant parameters.

### CIDP and diabetes

**Fig. 1:**
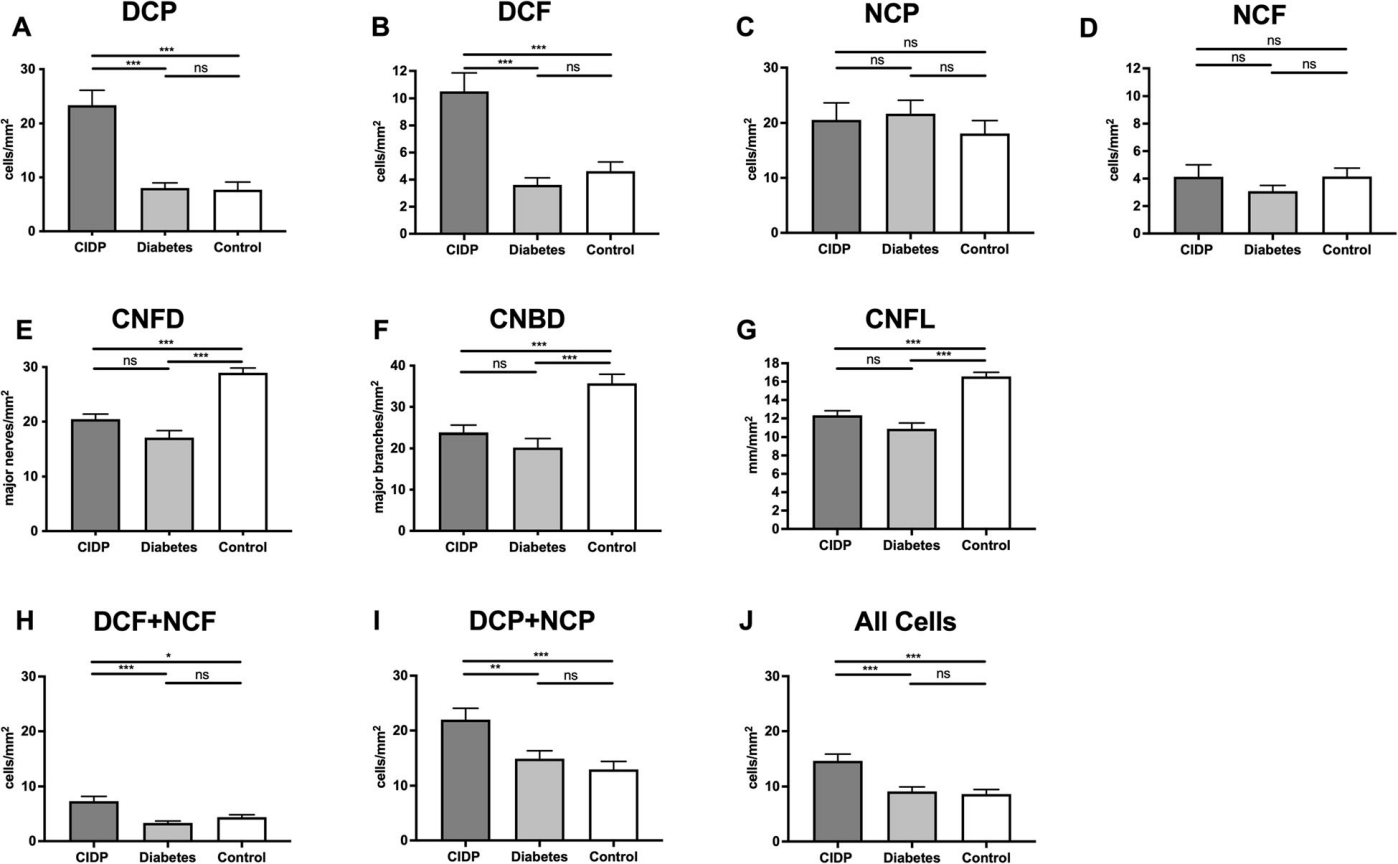
Corneal infiltrating cells and nerve fiber parameters in participants with chronic inflammatory demyelinating polyneuropathy (CIDP) or diabetes. Corneal cellular infiltrates were classified as dendritic cells (without [DCP] or with [DCF] fiber contact) or non-dendritic cells (without [NCP] or with [NCF] fiber contact) in participants with CIDP, diabetes and control participants **a**–**d**. Corneal nerve fiber density (CNFD), corneal nerve branch density (CNBD), and corneal nerve fiber length (CNFL) were quantified **e**–**g**. The number of infiltrating cells with proximity to nerve fibers **h**, the number of cells without nerve fiber contact **i**, and the total corneal cell count **j** were determined. Mean ± SEM, *P < 0.05, **P < 0.01, ***P < 0.001, ns indicates not significant

Patients with CIDP had a significantly higher DCP and DCF compared to patients with diabetes and healthy controls (Fig. 1a, b). There were no significant differences among groups for NCP (Fig. 1c) or NCF (Fig. 1d). However, all corneal nerve fiber parameters (CNFD, Fig. 1e; CNBD, Fig. 1f; CNFL, Fig. 1g) were significantly reduced in patients with CIDP and diabetes compared to controls, with no significant difference between CIDP and diabetes. The number of infiltrating cells in proximity to nerve fibers (DCF + NCF, Fig. 1h), the number of cells without nerve fiber contact (DCP + NCP, Fig. 1i), and the total cell number (Fig. 1j) was significantly higher in patients with CIDP compared to patients with diabetes or compared to control.

To test whether the detection of CIDP could be further improved, as per pre-specified analysis, we normalized infiltrating cell numbers to nerve fiber parameters. For the ratio of total cell numbers to nerve fiber parameters, there was no significant improvement in identifying CIDP (Supplementary Figure 1a-c). There was a significant difference for cells in proximity to nerve fibers (DCF + NCF) and their ratio for CNFD, CNBD, and CNFL between patients with CIDP and diabetes (Supplementary Figure 1D-F). There was no significant difference for cells in the periphery (DCP + NCP) and their ratio to nerve fiber parameters between patients with CIDP and diabetes (Supplementary Figure 1g-i).

By comparing effect sizes (*d*), the ratio of DCF + NCF to CNFL was identified as the most selective (F/mm^2^/CNFL *d* = 0.857), followed by F/mm^2^/CNFD (*d* = 0.664), F/mm^2^/CNBD (*d* = 0.408), P/mm^2^/CNFL (*d* = 0.225), P/mm^2^/CNFD (*d* = 0.083), and P/mm^2^/CNBD (*d* = 0.079). However, these derived parameters did not improve the ability to distinguish CIDP from DN beyond the number of infiltrating dendritic cells, which showed the highest effect size (DCF/mm^2^: *d* = 1.014; DCP/mm^2^: *d* = 0.941). DCP distinguished CIDP from DN with a sensitivity of 0.701, specificity of 0.879, and a PPV of 0.784. DCF distinguished CIDP from DN with a sensitivity of 0.596, specificity of 0.775, and a PPV of 0.723. For distinguishing CIDP from healthy control, DCP showed a sensitivity of 0.701, specificity of 0.767, and a PPV of 0.754, while DCF showed a sensitivity of 0.596, specificity of 0.722, and a PPV of 0.693.

### Patients with and without diabetic neuropathy

**Fig. 2:**
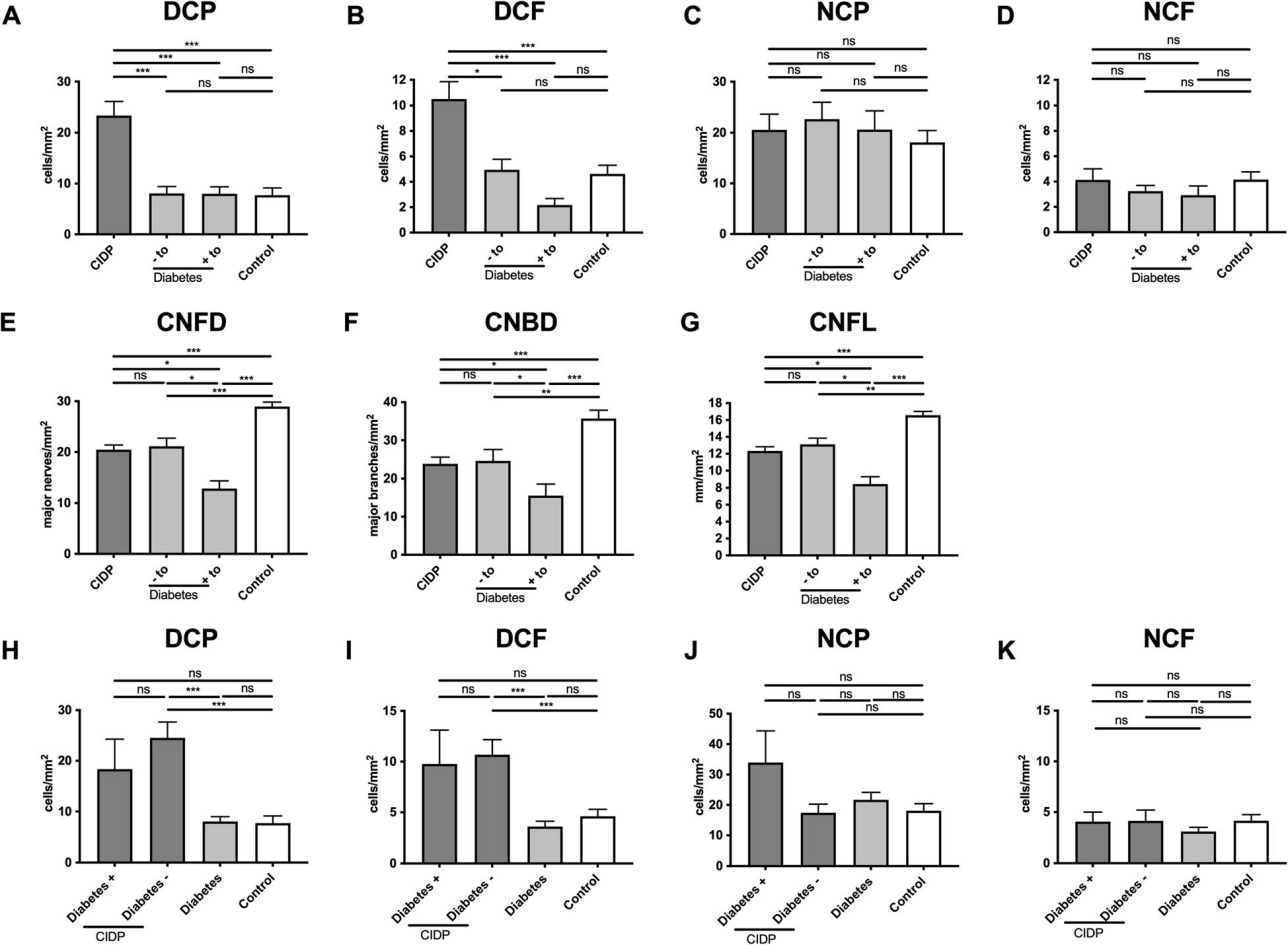
Corneal infiltrating cells and nerve fiber parameters in participants with chronic inflammatory demyelinating polyneuropathy (CIDP) and diabetic neuropathy. Corneal confocal microscopy was used to classify corneal cellular infiltrates as dendritic cells (without [DCP] or with [DCF] fiber contact) or non-dendritic cells (without [NCP] or with [NCF] fiber contact) and to assess corneal nerve fiber density (CNFD), corneal nerve branch density (CNBD), and corneal nerve fiber length (CNFL) in participants with diabetes who fulfilled (+to) or did not fulfill (−to) the Toronto criteria for large fiber neuropathy, in participants with diabetes and in healthy individuals (control) **a**–**g**. Participants with CIDP were further subdivided into those with (+) or without (−) diabetes **h**–**k**. Mean ± SEM, *P < 0.05, **P < 0.01, ***P < 0.001, ns indicates not significant

Patients with diabetes were further subdivided into patients with (+to) and without (−to) neuropathy according to the Toronto criteria. Both +to and −to had significantly lower numbers of DCP and DCF compared to patients with CIDP (Fig. 2a, b). There were no significant differences between groups for NCP and NCF (Fig. 2c, d). CNFD, CNBD and CNFL were lower in +to compared to −to, and CNFD, CNBD and CNFL were lower in −to group compared to controls (Fig. 2e-g).

### Influence of glycemia

There was no significant difference in corneal cellular infiltrates in a comparison of participants with poor versus good glycemic control (HbA_1c_ > 7.0% vs. ≤ 7.0%, Supplementary Figure 1j-k).

### CIDP and diabetes

There were no significant differences in DCP, DCF, NCP, and NCF in a comparison of participants with CIDP with and without diabetes (Fig. 2h–k). DCP was significantly higher in the CIDP subgroup without diabetes but not in the CIDP subgroup with diabetes compared to participants with diabetes alone (Fig. 2h). CIDP individuals without diabetes had significantly higher numbers of DCF compared to individuals with diabetes alone (Fig. 2i). There were no significant differences for NCP and NCF (Fig. 2j, k) as well as for CNFD, CNFL, and CNBD in CIDP participants with or without diabetes (data not shown).

### MGUS neuropathy

**Fig. 3:**
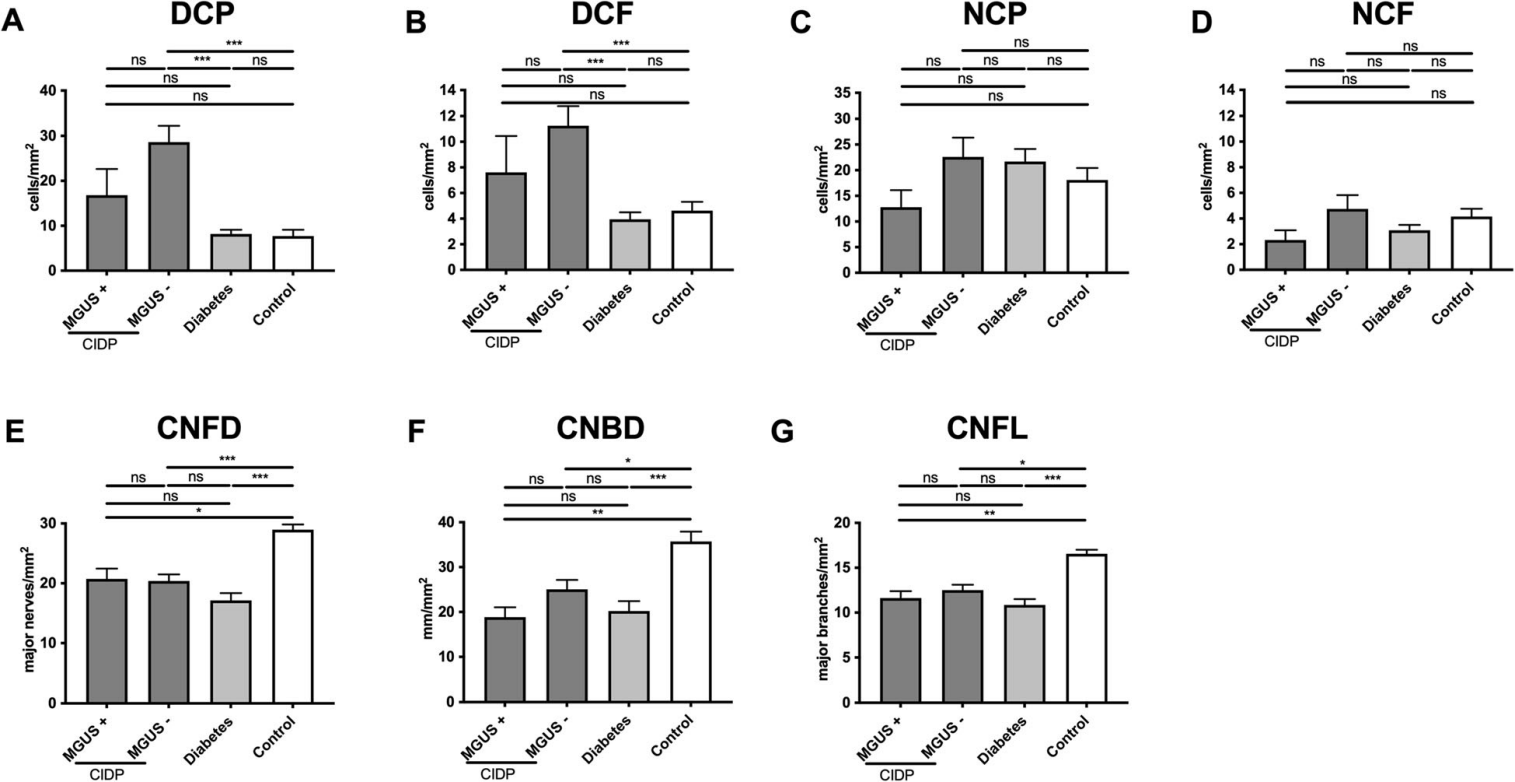
Corneal infiltrating cells and nerve fiber parameters in participants with chronic inflammatory demyelinating polyneuropathy (CIDP) with (+), or without (−) monoclonal gammopathy of undetermined significance (MGUS). Corneal confocal microscopy was used to classify corneal cellular infiltrates as dendritic cells (without [DCP] or with [DCF] fiber contact) or non-dendritic cells (without [NCP] or with [NCF] fiber contact) in participants with CIDP with (+) or without (−) MGUS, in participants with diabetes, and control individuals **a**–**d**. Corneal nerve fiber density (CNFD), corneal nerve fiber length (CNFL), and corneal nerve branch density (CNBD) were quantified **e**–**g**. Mean ± SEM, **P < 0.01, ***P < 0.001, ns indicates not significant

Participants with CIDP were divided into those with (*n* = 11) and without (*n* = 44) MGUS (Fig. 3). Dendritic cell density (DCP and DCF) was significantly higher in the CIDP subgroup without MGUS compared to the diabetes or control groups (Fig. 3a, b). No significant differences were observed among any of the groups for NCP and NCF (Fig. 3c, d). CNFD, CNFL, and CNBD were significantly lower in the MGUS+, MGUS− and diabetes groups compared to controls and did not differ between MGUS+ and MGUS− groups (Fig. 3e–g).

## Discussion

Up until now, there has been no non-invasive method available to assess ongoing inflammation in the peripheral nervous system. Therefore, we aimed to investigate the potential of CCM to distinguish inflammatory from non-inflammatory neuropathies. We show an increase in corneal dendritic cells in proximity to corneal nerve fibers in participants with CIDP compared to diabetic neuropathy.

The prevalence of CIDP is increased among individuals with diabetes [7, 28]. However, the development of CIDP in persons with diabetes is often not diagnosed or misdiagnosed as diabetic neuropathy and these individuals may be denied timely and effective treatment [3]. None of the known biomarkers or diagnostic tools such as cerebrospinal fluid protein, clinical, electrophysiological, and serological markers of autoimmunity can adequately identify CIDP in persons with demyelinating neuropathy due to another condition such as diabetic neuropathy. Significant proximal motor involvement without autonomic involvement [29] and electrophysiological slowing may help to establish the diagnosis of CIDP as opposed to diabetic neuropathy [30–34]. Sural nerve biopsies can be used to identify inflammation but remain impractical as they are invasive and, therefore, of limited use in clinical practice [35, 36]. Immunohistochemical studies of cells in the cornea using cell-specific surface markers [37] indicate that the corneal dendritic cells are mainly Langerhans cells [38, 39] and a previous study has shown that contact between these dendritic cells and corneal nerves may trigger nerve fiber damage [22].

Our previous study showed corneal nerve loss and an increase in dendritic cells in proximity to corneal nerve fibers, which was associated with the severity of motor symptoms in CIDP [23].

Here, we show moderate diagnostic utility of CCM identification of increased corneal dendritic cell density to differentiate CIDP from diabetic neuropathy. This study also excluded HbA_1c_ as a confounder in detecting CIDP among individuals with diabetes.

CCM was also useful to detect CIDP in those individuals with and without coexisting diabetes, indicating that the increase in dendritic cells is driven by peripheral nerve inflammation. Indeed, recent longitudinal studies have shown that increased dendritic cell infiltration may predict worsening outcomes in CIDP [24] and infiltrates may be reduced in response to immune therapy [25].

Our data also show that dendritic cell density differs in CIDP subgroups with and without MGUS supporting the assertion that persons with CIDP and MGUS have different clinical and electrophysiological patterns and underlying pathophysiological mechanisms [40–42]. These findings are in keeping with our previous study where we also showed equivalent corneal nerve loss but lower numbers of corneal dendritic cells in those with MGUS compared to CIDP [23].

## Conclusion

Our study shows that corneal confocal microscopy, by enabling the quantification of corneal dendritic cells, may have clinical utility to differentiate inflammatory from diabetic neuropathy. However, larger longitudinal studies are required to evaluate the potential of this method to predict disease progression and response to treatment.

## Supplementary Information

**Additional file 1: Supplement Figure 1**. Ratios of Corneal Infiltrating Cells to Nerve Fiber Parameters in Participants with Chronic Inflammatory Demyelinating Polyneuropathy (CIDP) or Diabetes and Effect of Glycemia on Corneal Infiltrating Cells. In participants with CIDP or diabetes and controls, ratios were calculated for (a-c) total corneal cell counts to corneal nerve fiber density (CNFD), corneal nerve branch density (CNBD), and corneal nerve fiber length (CNFL); (d-f) DCF + NCF (total number of infiltrating cells with proximity to nerve fibers) to CNFD, CNBD, and CNFL; and (g-i) DCP + NCP (total number of cells without nerve fiber contact) to CNFD, CNBD, and CNFL. All participants were stratified into two groups based on glycated hemoglobin (HbA_1c_) levels: >7.0% and ≤7.0% and corneal infiltrating cells were quantified (j-l). Mean ± SEM, *P<0.05, **P<0.01, ***P<0.001, ns indicates not significant.

## Data Availability

The datasets used and/or analyzed during the current study are available from the corresponding author on reasonable request.
