# Supplementary figures and images for: Corneal confocal microscopy differentiates inflammatory from diabetic neuropathy
Source: J Neuroinflammation. 2021 Apr 8;18:89. doi: 10.1186/s12974-021-02130-1 (PMC8033689; doi:10.1186/s12974-021-02130-1)

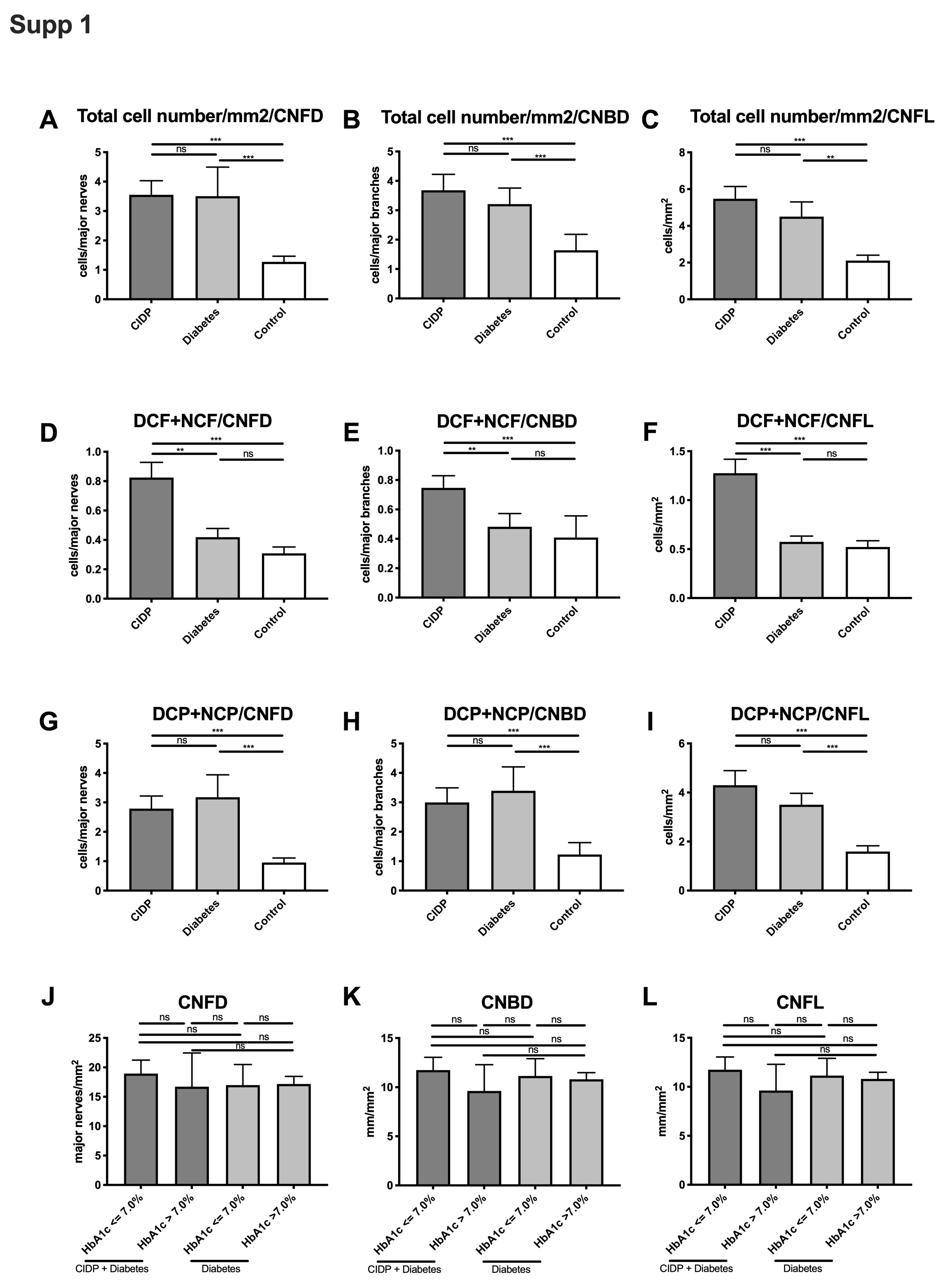

Supplement: Supplementary file 1 — Additional file 1: Supplement Figure 1. Ratios of Corneal Infiltrating Cells to Nerve Fiber Parameters in Participants with Chronic Inflammatory Demyelinating Polyneuropathy (CIDP) or Diabetes and Effect of Glycemia on Corneal Infiltrating Cells. In participants with CIDP or diabetes and controls, ratios were calculated for (a-c) total corneal cell counts to corneal nerve fiber density (CNFD), corneal nerve branch density (CNBD), and corneal nerve fiber length (CNFL); (d-f) DCF + NCF (total number of infiltrating cells with proximity to nerve fibers) to CNFD, CNBD, and CNFL; and (g-i) DCP + NCP (total number of cells without nerve fiber contact) to CNFD, CNBD, and CNFL. All participants were stratified into two groups based on glycated hemoglobin (HbA1c) levels: >7.0% and ≤7.0% and corneal infiltrating cells were quantified (j-l). Mean ± SEM, *P<0.05, **P<0.01, ***P<0.001, ns indicates not significant. [file 12974_2021_2130_MOESM1_ESM.tiff]
